# Association between serum uric acid and major chronic diseases among centenarians in China: based on the CHCCS study

**DOI:** 10.1186/s12877-021-02185-y

**Published:** 2021-04-07

**Authors:** Fuyin Kou, Shanshan Yang, Shengshu Wang, Miao Liu, Yao He

**Affiliations:** 1 Health Service Department, Chinese PLA General Hospital, Beijing, China; 2 Department of Disease Prevention and Control, The 1st Medical Center, Chinese PLA General Hospital, Beijing, China; 3 Beijing Key Laboratory of Aging and Geriatrics, National Clinical Research Center for Geriatrics Diseases, State Key Laboratory of Kidney Diseases, Institute of Geriatrics, Second Medical Center of Chinese PLA General Hospital, Beijing, China; 4 Graduate School of Chinese PLA General Hospital, Institute of Geriatrics, Chinese PLA General Hospital, 28 Fuxing Road, Beijing, 100853 China

**Keywords:** Serum uric acid, Hyperuricemia, Dyslipidemia, Centenarian

## Abstract

**Background:**

This study aims to analyze the distribution of serum uric acid (SUA) level based on more than 1000 centenarians and to explore the association with three common diseases including hypertension, diabetes and dyslipidemia.

**Methods:**

All the 1002 centenarians from the CHCCS were included. Household survey was conducted.

**Results:**

The mean SUA level of centenarians was 329.04 ± 97.75 μmol/L and the prevalence of hyperuricemia in centenarians was 26.5%. There was no statistical difference in the distribution of SUA levels among centenarians with or without hypertension/diabetes. For dyslipidemia, there was an independent positive association. The risk of dyslipidemia among those with hyperuricemia was 1.646 (95%CI: 1.078–2.298) compared with those who didn’t have hyperuricemia. By comparing different subtypes of dyslipidemia, hyperuricemia was positively associated with hypertriglyceridemia and low-density lipoprotein cholesterolemia, with the corresponding ORs of 2.553 (95%CI: 1.282–5.083) and 1.927 (95%CI: 1.273–2.917) respectively, while there was no statistically significant association with hypercholesterolemia 0.998 (95%CI: 0.574–1.732).

**Conclusions:**

There was no relation between SUA with hypertension or diabetes, while there was independently and positively association with hypertriglyceridemia and low-density lipoprotein cholesterolemia. The health benefits of controlling SUA in centenarians still require evidence based on prospective studies.

**Supplementary Information:**

The online version contains supplementary material available at 10.1186/s12877-021-02185-y.

## Background

Serum uric acid (SUA) is an important index of renal function. It is the product of protein metabolism [1]. The current physiological reference value of SUA was mainly from data about adults [2–5]. However, it is well known that kidney function deteriorates with age. The renal function of centenarians is much worse than that of adults or young elderly. But there is little data on theSUA level about this extremely survivors [6, 7]. It ‘s still unknown whether it is the same as the general elderly, or as a template for healthy aging.

On the other hand, SUA is not only an indicator of renal function, but also an important risk factor for cardiovascular disease and metabolic diseases. Meta-analysis showed that SUA was a risk factor for hypertension, metabolic syndrome, and coronary heart diseases [8, 9]. Therefore, SUA has become one of the early warning and intervention indicators of major chronic diseases. However, most of the current evidence was based on adults, or younger elderly aged < 80 years old. There was a lack of basic data for centenarians. Whether this trend still exists among centenarians is unknown. That is to say, in centenarians, whether SUA is still an important risk factor for major diseases and needs to be controlled, there is no sufficient evidence yet. So, this study used data from one of Asia’s largest centenarian survey to analyze the distribution of SUA level based on more than 1000 centenarians and to explore the association with three common diseases including hypertension, diabetes and dyslipidemia.

## Methods

### Study population

All the subjects in this study were from CHCCS, and the detailed research design framework was shown in previous articles [10]. In brief, this was a full sample survey. Based on the demographic data of 18 cities of Hainan Province provided by the Civil Affairs Bureau, we included all the people aged 100 and over in the study. A total of 1002 centenarians were invited to the survey and included in the analysis.

### Investigation method

Household survey was conducted. All the questions on the questionnaire were asked and recorded by trained investigators. Centenarians were asked to answer health-related questions themselves. For those who were unable to answer the questions, the caregivers or relatives should answer on their behalf. All investigators were medical staff from Hainan hospital of Chinese PLA general hospital, who have received unified training. A unified questionnaire was used to collect detailed demographic characteristics, disease history, family history, and lifestyles. Measurements included height (For those with marijuana, measure their length), weight, waist circumference (WC), and blood pressure. Elbow vein blood was taken at 7–8 a.m. (more than 8 h of fasting), and sent to the biochemical department of Hainan hospital of Chinese PLA general hospital to detect the relevant biochemical indicators, including total cholesterol (TC), triglycerides (TG), high-density lipoprotein cholesterol (HDL-C), low-density lipoprotein cholesterol (LDL-C), fasting blood glucose (FPG), albumin (ALB), serum uric acid (SUC), creatinine (Scr), blood urea nitrogen (BUN).

### Definitions

Centenarians referred to those who have reached the age of 100 years old at the survey. Age was calculated as the date of investigation minus the date of birth (checked according to identification card and the certificate of Hainan Civil Affairs Bureau) [10]. Body mass index (BMI) was calculated as weight (in kilograms) divided by the square of height (in meters).

Hyperuricemia was defined as positive if SUA level ≥ 420 μmol/L in men or ≥ 360 μmol/L in women or previous diagnosed [11, 12].

Abdominal obesity was defined as positive if WC ≥ 90 cm in men or WC ≥ 85 cm in women [13].

Blood pressure was classified into the following categories: normal blood pressure: no history of hypertension in the past and SBP < 120 and DBP < 80 mmHg; prehypertension: no history of hypertension in the past and 120 mmHg < SBP < 140 mmHg or 80 mmHg < DBP < 90 mmHg; Hypertension, previous diagnosis or SBP ≥ 140 mmHg or DBP ≥ 90 mmHg [14].

Blood glucose was classified into the following categories: normal blood glucose, no history of diabetes in the past and FPG ≥ 6.1 mmol/L; Impaired fasting glucose (IFG), no history of diabetes in the past and 6.1 mmol/L ≤ FPG < 7.0 mmol/L; Diabetes, previous diagnosis or FPG ≥ 7.0 mmol/L [15].

Blood lipids was classified into the following categories: normal blood lipids, no history of dyslipidemia in the past and total cholesterol (TC) < 5.18 mmol/L, triglyceride (TG) < 1.70 mmol/L, high density lipoprotein cholesterol (HDL-C) ≥ 1.04 mmol/L, and low density lipoprotein cholesterol (LDL-C) < 3.37 mmol/L; elevated blood lipids: no history of dyslipidemia in the past and 5.18 mmol/L ≤ TC < 6.22 mmol/L, 1.70 mmol/L ≤ TG < 2.26 mmol/L, and 3.37 mmol/L ≤ LDL-C < 4.14 mmol/L; dyslipidemia, previous diagnosis or TC ≥ 6.22 mmol/L, or TG ≥ 2.26 mmol/L, or HDL-C < 1.04 mmol/L, or LDL-C ≥ 4.14 mmol/L. The subtypes of dyslipidemia were classified into the following categories: Hypercholesterolemia, TC ≥ 6.22 mmol/L; Hypertriglyceridemia, TG ≥ 2.26 mmol/L; Combined hyperlipidemia, TC ≥ 6.22 mmol/L and TG ≥ 2.26 mmol/L; low-density lipoprotein cholesterolemia, HDL-C < 1.04 mmol/L [16].

#### Covariates

Other related covariates included sex, age, ethnicity, education, marriage, previous work type, smoking, drinking and dietary habits. The categories of covariates were listed in Additional file 1: Table 1.

### Patient and public involvement

All centenarians were from Hainan Province. The investigation team obtained the list of centenarians from the Civil Affairs Department, and conducted household survey on those who agreed to participate in the survey. Face-to-face questionnaire survey was used tocollect health-related information of the centenarians. All centenarians were informaed and understood the design and purpose of the study, and participated in the study after signing the informed consent. The results of laboratory test and physical examination should be fed back to the elderly in time.

### Statistical analysis

Mean ± standard deviation (SD) was used for continuous variables and n(%) was uased for categorical variables. Analysis of variance was used to compare continuous variables between different groups, and chi-square test was used to compare categorical variables. Pearson correlation coefficients were used to describe correlations between SUA levels and other variables. Multivariable logistic regression was used to calculate the odds ratio (OR) of SUA for related diseases. The related potential factors with *p* < 0.10 in univariate analysis and those proved to be assocaited with dependent variables were included in the model. The forward stepwise (likelihood ratio) method was used to calculated the adujsted ORs. SUA was used as independent variableas in two forms: continuous value and binary variable (hyperuricemia) in the model. Three chronic diseases were used as dependent variable separately, including hypertension, diabetes and dyslipidemia. We also used pre-hypertension/hypertension, IFG/diabetes, elevated lipids/dyslipidemia as dependent variables in the sensitivity analysis. All the analysis was conducted in SPSS 20.0(SPSS Inc., Chicago, IL). All statistical tests were two sided, and *P* < 0.05 was considered statistically significant.

## Results

The mean age was 102.77 ± 2.55 years, with 82.0% were female. WC, TG, and HDL-C were related with SUA levels (*p* < 0.05). Correspondingly, there were statistical differences with abdominal obesity and dyslipidemia prevalence (Table 1).

**Table 1:** General characteristics of centenarians

Characteristics	Hyperuricemia		
	Yes	No	p
mean ± SD			
Age (yrs)	102.79 ± 3.01	102.76 ± 2.65	0.853
Weight (kg)	38.72 ± 8.18	37.17 ± 7.31	0.004
WC (cm)	77.01 ± 8.95	74.53 ± 8.74	< 0.001
BMI (kg/m^2^)	18.37 ± 3.47	17.92 ± 3.37	0.271
SBP (mmHg)	154.05 ± 25.75	152.03 ± 23.93	0.247
DBP (mmHg)	75.01 ± 12.68	76.02 ± 13.03	0.276
TC (mmol/l)	4.64 ± 0.99	4.69 ± 0.99	0.459
TG (mmol/l)	1.27 ± 0.75	1.14 ± 0.61	0.005
HDL-C (mmol/l)	1.39 ± 0.43	1.45 ± 0.37	0.041
LDL-C (mmol/l)	2.81 ± 0.82	2.81 ± 0.78	0.926
FPG (mmol/l)	5.18 ± 1.46	5.10 ± 1.44	0.466
ALB(g/L)	38.42 ± 3.88	38.52 ± 4.05	0.734
SUA (μmol/L)	450.04 ± 76.59	285.31 ± 60.69	< 0.001
n (%)			
Sex			0.247
Male	54 (30.0)	126 (70.0)	
Female	212 (25.8)	610 (74.2)	
Nationality			0.330
Han	230 (26.0)	653 (74.0)	
Minority	36 (30.3)	83 (69.7)	
Marriage status			0.343
Widowed	217 (26.0)	619 (74.0)	
Married/divorced/others	49 (29.5)	117 (70.5)	
Education level			0.880
Illiteracy	241 (26.3)	674 (73.7)	
Primary school	19 (28.4)	48 (71.6)	
Middle school or above	6 (30.0)	14 (70.0)	
Work types			0.410
Mental work	8 (34.8)	15 (65.2)	
Light or moderate physical labor	148 (27.6)	388 (72.4)	
Heavy physical labor	110 (24.8)	333 (75.2)	
Smoking			0.665
Never smoking	236 (26.3)	660 (73.7)	
Ever or current smoking	30 (28.3)	76 (71.7)	
Alcohol drinking			0.086
Never alcohol drinking	213 (25.5)	623 (74.5)	
Ever or current alcohol drinking	53 (31.9)	113 (68.1)	
Vegetables(≥1 time/day)			0.641
Yes	236 (26.8)	645 (73.2)	
No	30 (24.8)	91 (75.2)	
Fruits(≥1 time/day)			0.335
Yes	41 (29.9)	96 (70.1)	
No	225 (26.0)	640 (74.0)	
Meat(≥1 time/day)			0.568
Yes	92 (25.5)	269 (74.5)	
No	174 (27.1)	467 (72.9)	
Fish(≥1 time/day)			0.397
Yes	81 (24.8)	245 (75.2)	
No	185 (27.4)	491 (72.6)	
Eggs(≥1 time/day)			0.054
Yes	26 (27.4)	69 (72.6)	
No	250 (27.6)	657 (72.4)	
Milk(≥1 time/day)			0.774
Yes	31 (27.7)	81 (72.3)	
No	235 (26.4)	655 (73.6)	
Abdominal obesity			0.002
Yes	81 (34.2)	156 (65.8)	
No	185 (24.2)	580 (75.8)	
Hypertension			0.222
Yes	206 (27.5)	542 (72.5)	
No	60 (23.6)	194 (76.4)	
Dyslipidemia			0.001
Yes	72 (35.6)	130 (64.4)	
No	194 (24.3)	606 (75.8)	
Diabetes			0.272
Yes	30 (31.3)	66 (68.8)	
No	236 (26.0)	670 (74.0)	

### Distribution of SUA levels and prevalence of hyperuricemia

The mean SUA level of centenarians was 329.04 ± 97.75 μmol/L. 45.7% of them were between 250 and 350 μmol/L and 81.4% were between 200 and 450 μmol/L. Only 5.7% were under 200 μmol/L and 11.0% were > 450 μmol/L (Additional file 1: Table 2).

The mean SUA level in male was higher than in female (379.86 ± 110.08 μmol/L vs. 317.91 ± 9.19 μmol/L, *p* < 0.001). Compared with different age groups, the mean SUA levels were basically the same for each 5 years’ age group (100–104, 105–109, 110-years old). In other words, SUA levels didn’t increase or decrease with age.

The prevalence of hyperuricemia in centenarians was 26.5%. Male had a relatively higher prevalence than that of female, although not statistically significant (30.0% vs. 25.8%, *p* = 0.247). Similarly, there was no age-related trend (*p* = 0.361) (Table 2).

**Table 2:** Distribution of SUA levels and hyperuricemia prevalence

	Male	Female	p	Total
SUA (μmol/L)				
Age groups				
100–104 yrs	379.18 ± 113.48	319.29 ± 89.34	< 0.001	330.59 ± 97.16
105–109 yrs	383.27 ± 92.75	311.11 ± 100.28	< 0.001	323.41 ± 102.46
≥ 110 yrs	–	321.39 ± 85.96		321.39 ± 85.96
P	0.853	0.606		0.615
Total	379.86 ± 110.08	317.91 ± 91.19		329.04 ± 97.75
Hyperuricemia [n(%)]				
Age groups				
100–104 yrs	42 (28.0)	170 (26.4)	0.682	212 (26.7)
105–109 yrs	12 (40.0)	31 (21.2)	0.059	43 (24.4)
≥ 110 yrs	–	11 (35.5)	–	11 (35.5)
P	0.190	0.356		0.361
Total	54 (30.0)	212 (25.8)	0.247	266 (26.5)

### Correlation analysis of SUA levels and three common diseases

As can be seen from Table 3, SUA level was positively correlated with Scr and BUN level, with the Pearson’s correlation coefficients 0.528 and 0.389 respectively (*p* < 0.05). In addition, SUA level was positively correlated with obesity related indicators, including weight and WC, with the Pearson’s correlation coefficients of 0.228 and 0.188 respectively (*p* < 0.05). The Pearson’s correlation coefficients of SUA with SBP, DBP, blood glucose were − 0.003, − 0.056 and − 0.001 respectively (*p* > 0.05). On the other hand, SUA level was related with blood lipid, including TG (*r* = 0.119, *p* < 0.05) and HDL-C (*r* = − 0.101, *p* < 0.05), but not with TC(*r* = − 0.006, P 0.847) and LDL-C (*r* = 0.027, *p* = 0.401).

**Table 3:** Correlation analysis

Vararibles	r	p
Age (yrs)	−0.020	0.529
Weight (kg)	0.228	< 0.001
WC (cm)	0.188	< 0.001
BMI (kg/m^2^)	0.114	< 0.001
SBP (mmHg)	−0.003	0.918
DBP (mmHg)	−0.056	0.076
TC (mmol/l)	− 0.006	0.847
TG (mmol/l)	0.119	< 0.001
HDL-C (mmol/l)	−0.101	< 0.001
LDL-C (mmol/l)	0.027	0.401
FPG (mmol/l)	−0.001	0.967
ALB(g/L)	0.048	0.132

### Association between SUA levels and three common diseases

There was no statistical difference in the distribution of SUA levels among centenarians with or without hypertension. The mean SUA level for the three groups (normal blood pressure, pre-hypertension and hypertension) were 335.30 ± 91.33 μmol/L, 321.53 ± 87.46 μmol/L and 330.30 ± 100.69 μmol/L *p* = 0.473) respectively. The situation was similar to diabetes. The mean SUA level for the three groups (normal blood glucose, IFG and diabetes) were 328.84 ± 95.58 μmol/L, 329.35 ± 87.13 μmol/L, 330.49 ± 122.62 μmol/L, *p* = 0.987). There was statistical difference among different blood lipid groups. The mean SUA level for the three groups (normal blood lipids, elevated blood lipids and dyslipidemia) were 311.87 ± 90.91 μmol/L, 325.19 ± 89.63 μmol/L and 357.2 ± 116.92 μmol/L, *p* < 0.001) respectively.

Table 4 showed the result of multivariable logistic regression analysis of the association between SUA level and three common diseases. Hypertension, diabetes and dyslipidemia were used as dependent variables separately; SUA level was used as the independent variable; the related potential factors with *p* < 0.10 in univariate analysis and those proved to be assocaited with dependent variables were included in the model. The forward stepwise (likelihood ratio) method was used to calculated the adujsted ORs. As can be seen from Table 4, SUA levels was not associated with hypertension, the ORs were 1.000 (95%CI: 0.999–1.002) and 1.221 (95%CI: 0.874–1.707) when the independent SUA levels as continuous variable and dichotomous variable (hyperuricemia) respectively. Similarly, SUA levels was not associated with diabetes either, the ORs were 1.000 (95%CI: 0.998–1.002) and 1.290 (95%CI: 0.818–2.037) respectively. For dyslipidemia, there was an independent positive association. Compared with those who didn’t have hyperuricemia, the risk of dyslipidemia among those with hyperuricemia were 1.646 (95%CI: 1.078–2.298). By comparing different subtypes of dyslipidemia, hyperuricemia was positively associated with hypertriglyceridemia and low-density lipoprotein cholesterolemia, with the corresponding ORs of 2.553 (95%CI: 1.282–5.083) and 1.927 (95%CI: 1.273–2.917) respectively, while there was no statistically significant association with hypercholesterolemia 0.998 (95%CI: 0.574–1.732).

**Table 4:** Multivariable analysis of SUA Level and major chronic diseases

Independent variables	SUA level (continuous, per μmol/L)		hyperuricemia (dichotomous)	
	OR(95%CI)	p	OR(95%CI)	p
Hypertension	1.000 (0.999–1.002)	0.286	1.221 (0.874–1.707)	0.242
Prehypertension & hypertension	1.000 (0.997–1.002)	0.578	1.115 (0.650–1.912)	0.691
Diabetes	1.000 (0.998–1.002)	0.879	1.290 (0.818–2.037)	0.273
IFG& diabetes	1.000 (0.998–1.002)	0.995	1.255 (0.878–1.793)	0.213
Dyslipidemia	1.003 (1.002–1.005)	< 0.001	1.646 (1.078–2.298)	0.003
Elevated BL& Dyslipidemia	1.001 (1.000–1.002)	0.142	1.201 (1.001–1.594)	0.024
Hypercholesterolemia	1.001 (0.999–1.004)	0.222	0.998 (0.574–1.732)	0.993
Hypertriglyceridemia	1.005 (1.002–1.008)	0.001	2.553 (1.282–5.083)	0.008
Combined hyperlipidemia	1.007 (1.002–1.011)	0.005	2.794 (0.694–11.252)	0.148
Low HDL-C	1.004 (1.002–1.006)	< 0.001	1.927 (1.273–2.917)	0.002

Adjusted for age, sex, nationality, education, marital status, BMI, work types, smoking, alcohol drinking, eating habits (vegetables, fruits, meat, fish, egg, milk), abdominal obesity

## Discussion

This result based on CHCCS showed that the prevalence of hyperuricemia in centenarians was high, and it had independently and positively association with hypertriglyceridemia and low density lipoprotein cholesterolemia while no association with hypertension or diabetes.

SUA level was higher than that of adults and younger elderly. The prevalence of hyperuricemia was also at a high level among centenarians. Previous study based on 22,983 Chinese adults based on Chinese Physiological Constant and Health Conditions Study showed that the average SUA level in Chinese population was 346.1umol/L and 258.6umol/L for males and females respectively. The prevalence of hyperuricemia was 13.0% among adult, while it went up to 20.5% among elderly [17]. The study based on elderly from Beijing showed the mean SUA levels were 345.1umol/L and 309.4umol/L in male and female respectively, and the prevalence of hyperuricemia was 16.7% [18]. One meta-analysis based on 38 studies showed that the prevalence of hyperuricemia in Chinese people was 13.3%. This study showed that the mean SUA level was 329.04 μmol/L in centenarians, which was higher than previous studies based on adults or younger elderly [12]. Male had slightly higher SUA levels than female, but there was no statistical difference, which was consistent with previous results [13]. Meta-analysis showed that the sex difference in the prevalence of hyperuricemia narrowed among people over 50 years old [19]. Part of the reason is that postmenopausal women lose their protection with the decrease of estrogen.

There was no relation between SUA with hypertension or diabetes, which was inconsistent with previous studies based on adults or younger elderly. Meta-analysis based on 18 cohort studies with 55,607 people showed that the risk of hypertension in patients with hyperuricemia was 1.41(95%CI: 1.23–1.58) [20, 21]. Another meta-analysis based on 16 cohort study with 61,714 people showed that the risk of diabetes in patients with hyperuricemia was 1.131(95%CI: 1.084–1.179) [22]. However, the above meta-studies only included data of adults and people less than 80, and lacked data on centenarians. The association between SUA and diabetes or hypertension tends to weaken with the increase of age. The results from the Project of Longevity and Aging in Dujiangyan study also showed that SUA was not associated with hypertension based on 870 nonagenarians/centenarians [6]. Results from Health Professionals Follow-Up Study also showed similar results based on elderly men aged 60–85 [23]. These results were consistent with our conclusion. On the other hand, this study showed that SUA was independently and positively correlated with hypertriglyceridemia and low-density lipoprotein cholesterolemia. A 5-year cohort study of 6476 adults from Japan showed that the risk of low-density lipoprotein cholesterolemia for every 1 mg/dL increase in SUA level was 1.159 (95% CI: 1.009–1.331) in male and 1.215 (95% CI: 1.061–1.390) in female respectively [24]. Another study based on the results of 8-year follow-up of 4190 adults, showed that the incidence of hypertriglyceridemia was 28.2, 29.1, 36.9 and 45.6% respectively with the quartile of SUA level [25]. Those results were consistent with this study, which was the first to analyze the association of SUA with dyslipidemia and the subtypes (including hypertriglyceridemia and low-density lipoprotein cholesterolemia) in centenarians. The results showed that there was a significant positive correlation between SUA and hypertriglyceridemia and low-density lipoprotein cholesterolemia.

Our study has several strengths. This is the largest centenarian study in Asia, which is very presentative. Secondly, unlike other sociological surveys which focus on sociology but pay less attention to health-related issues, this CHCCS survey was conducted by professional medical staff, which provided reliable and sufficient medical information. Third, we analyzed the association between SUA with all three common chronic diseases at the same time, which may be helpful for the comparisons.

On the other hand, this study had several limitations. First, this study was a cross-sectional study with limited causal inference, which still needs to be verified by prospective research. Second, SUA was a one-time test, which may be affected by diet and other factors. Third, lack of information about drug use may have an impact on the outcome. But, according to the Additional file 1: Table 3, there was less than 1/4 of the centenarians who were taking medicine. Fourth, there may be survivor bias; the centenarians surveyed were relatively healthy people, which may underestimate the risk.

## Conclusion

In summary, according to the results of CHCCS, which has the largest sample of centenarians, the SUA level in centenarians is higher and the prevalence of hyperuricemia is also at a higher level. SUA was not related to hypertension or diabetes, but positively correlated with hypertriglyceridemia and low-density lipoprotein cholesterolemia. The health benefits of controlling SUA in centenarians still requires evidence based on prospective studies.

## Supplementary Information

**Additional file 1: Table 1.** Characterics of covariates. **Table 2.** Distribution of SUA levels. **Table 3.** Distribution of the number of drugs taken.

## Data Availability

The datasets used during the current study are available on reasonable request. Please contact corresponding author Professor Liu (liumiaolmbxb@163.com).

## Abbreviations

ALB: Albumin
BL: Blood lipids
BMI: Body mass index
BUN: Serum urea nitrogen
CI: Confidence interval
CHCCS: China Hainan Centenarian Cohort Study
DBP: Diastolic blood pressure
FBG: Fasting plasma glucose
HDL-C: High density lipoprotein cholesterol
IFG: Impaired fasting glucose
LDL-C: Low density lipoprotein cholesterol
OR: Odds ratio
SBP: Systolic blood pressure
Scr: Serum creatinine
SUA: Serum uric acid
TC: Total cholesterol
TG: Triglyceride
