# Supplementary material for: Association between serum uric acid and major chronic diseases among centenarians in China: based on the CHCCS study
Source: BMC Geriatr. 2021 Apr 7;21:231. doi: 10.1186/s12877-021-02185-y (PMC8028058; doi:10.1186/s12877-021-02185-y)
Supplement: Supplementary file 1 — Additional file 1: Table 1. Characterics of covariates. Table 2. Distribution of SUA levels. Table 3. Distribution of the number of drugs taken. [file 12877_2021_2185_MOESM1_ESM.docx]

Appendix table1. Characterics of covariates

| Covariates | Catogory | Definition |
| --- | --- | --- |
| Sex | Categorical variables | Male, Female |
| Age | Continuous variable |  |
| Ethnicity | Categorical variables | Han, Minority |
| Education | Categorical variables | Illiteracy, Primary school, Middle school or above |
| Previous work type | Categorical variables | Mental work, Light or moderate physical labor, Heavy physical labor |
| Smoking | Categorical variables | Never smoking, Ever or current smoking |
| Alcohol drinking | Categorical variables | Never alcohol drinking, Ever or current alcohol drinking |
| Vegetables(≥1 time/day) | Categorical variables | Yes, No |
| Fruits(≥1 time/day) | Categorical variables | Yes, No |
| Meat(≥1 time/day) | Categorical variables | Yes, No |
| Fish(≥1 time/day) | Categorical variables | Yes, No |
| Eggs(≥1 time/day) | Categorical variables | Yes, No |
| Milk(≥1 time/day) | Categorical variables | Yes, No |

Appendix table2. Distribution of SUA levels

| SUA(μmol/L) | <200 μmol/L | 200-249μmol/L | 250-299μmol/L | 300-349μmol/L | 350-399μmol/L | 400-449μmol/L | ≥450μmol/L | p |
| --- | --- | --- | --- | --- | --- | --- | --- | --- |
| Sex |  |  |  |  |  |  |  | <0.001 |
| Male | 6(3.3) | 8(4.4) | 24(13.3) | 37(20.6) | 37(20.6) | 68(37.8) | 40(22.2) |  |
| Female | 51(6.2) | 134(16.3) | 178(21.7) | 219(26.6) | 103(12.5) | 137(16.7) | 70(8.5) |  |
| Age groups |  |  |  |  |  |  |  | 0.305 |
| 100-104 yrs | 41(5.2) | 112(14.1) | 158(19.9) | 203(25.5) | 117(14.7) | 164(20.6) | 88(11.1) |  |
| 105-109 yrs | 13(7.4) | 27(15.3) | 39(22.2) | 45(25.6) | 18(10.2) | 34(19.3) | 22(12.5) |  |
| ≥110 yrs | 3(9.7) | 3(9.7) | 5(16.1) | 8(25.8) | 5(16.1) | 7(22.6) | 0(0.0) |  |
| Total | 57(5.7) | 142(14.2) | 202(20.2) | 256(25.5) | 140(14.0) | 205(20.5) | 110(11.0) |  |

Appendix table3. Distribution of the number of drugs taken

| Number of drugs taken | n(%) |
| --- | --- |
| 0 | 774(77.24) |
| 1 | 144(14.37) |
| 2 | 46(4.59) |
| 3 | 30(2.99) |
| 4 | 4(0.40) |
| 5 | 3(0.30) |
| 6 | 1(0.10) |
